# Supplementary material for: Growth Temperature Influence on Atomic-Layer-Deposited In2O3 Thin Films and Their Application in Inorganic Perovskite Solar Cells
Source: Nanomaterials (Basel). 2021 Aug 11;11(8):2047. doi: 10.3390/nano11082047 (PMC8399107; doi:10.3390/nano11082047)
Supplement: Supplementary file 1 [file nanomaterials-11-02047-s001.zip › nanomaterials-1321435-supplementary.pdf]

# Supplementary Materials

# Growth Temperature Influence on Atomic-Layer-Deposited $In_2O_3$ Thin Films and Their Application in Inorganic Perovskite Solar Cells

**Umme Farva [1], Hyeong Woo Lee [1], RiNa Kim [1], Dong-Gun Lee [2], Dong-Won Kang [2,*] and Jeha Kim [1,*]**

[1] Department of Energy Convergence Engineering, Cheongju University, Cheongju 28503, Korea; ummefarva@gmail.com (U.F.); hwkj0224@gmail.com (H.W.L.); kimrina74@gmail.com (R.K.)
[2] School of Energy Systems Engineering, Chung-Ang University, Seoul 06974, Korea; padawan1215@naver.com
* Correspondence: jeha@cju.ac.kr (J.K.); kangdwn@cau.ac.kr (D.-W.K.)

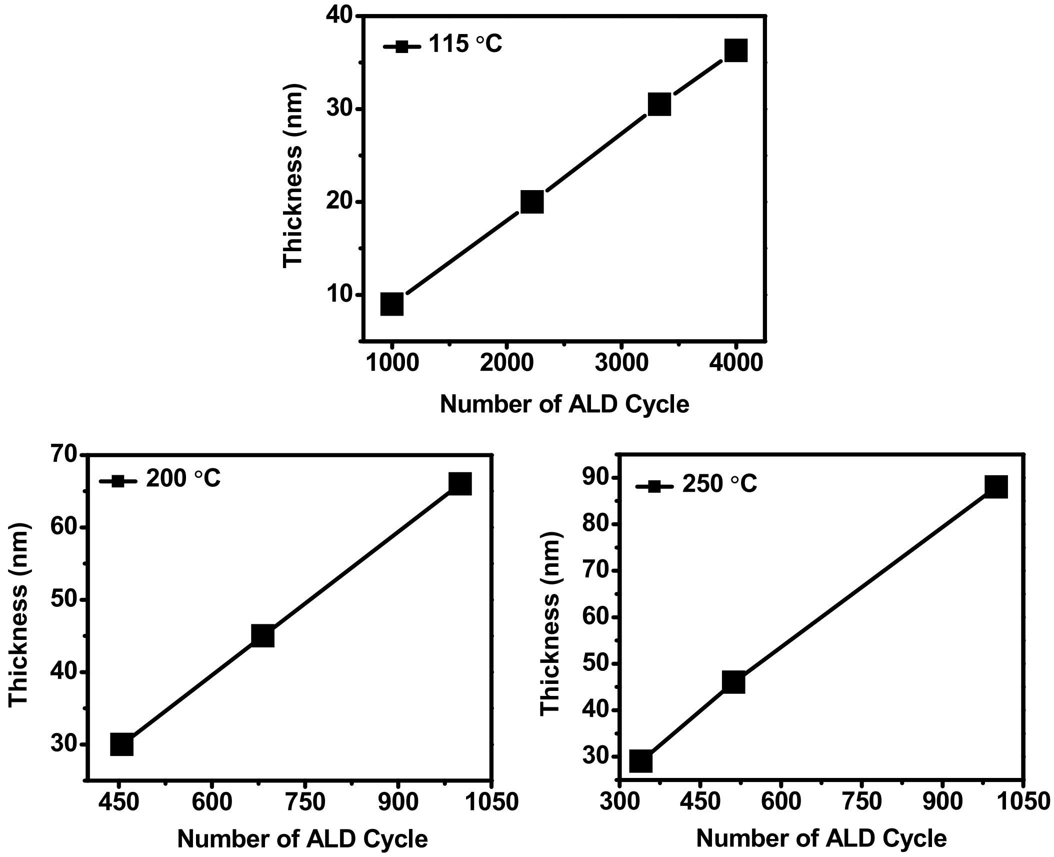


**Figure S1.** ALD deposited $In_2O_3$ film thickness as a function of the number of ALD cycles for film deposited at 115 °C, 200 °C and 250 °C on the Si substrate.

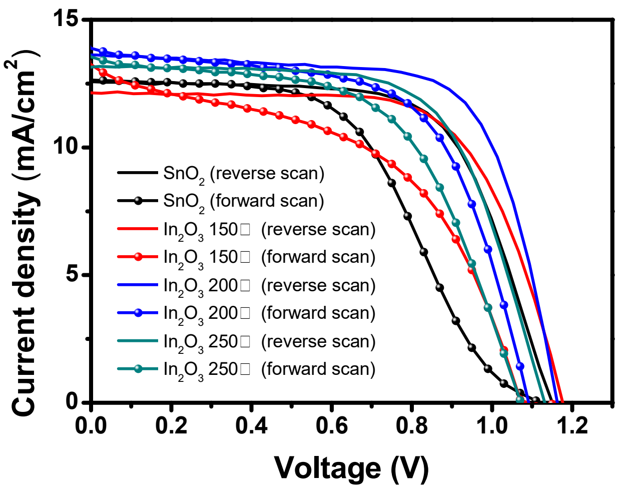


**Figure S2.** Hysteresis observation for developed $CsPbI_2Br$ perovskite solar cells with different ETLs.

**Table S1.** Photovoltaic parameters and hysteresis index of fabricated $CsPbI_2Br$ perovskite solar cells with different ETLs.

| Sample | $J_{SC}$ ($mA/cm^2$) | $V_{OC}$ (V) | FF (%) | PCE (%) | Hysteresis Index (HI) |
|---|---|---|---|---|---|
| **$SnO_2$ reverse scan** | 12.54 | 1.12 | 67.89 | 9.52 | 0.75 |
| **$SnO_2$ forward scan** | 12.62 | 1.11 | 50.76 | 7.11 | |
| **$In_2O_3$ 150°C reverse scan** | 13.18 | 1.10 | 67.37 | 9.77 | 0.85 |
| **$In_2O_3$ 150°C forward scan** | 13.41 | 1.07 | 58.06 | 8.33 | |
| **$In_2O_3$ 200°C reverse scan** | 12.13 | 1.15 | 68.54 | 9.53 | 0.74 |
| **$In_2O_3$ 200°C forward scan** | 12.92 | 1.07 | 50.71 | 7.01 | |
| **$In_2O_3$ 250°C reverse scan** | 13.64 | 1.13 | 70.99 | 10.97 | 0.85 |
| **$In_2O_3$ 250°C forward scan** | 13.75 | 1.07 | 62.99 | 9.27 | |
